# HPV16-E7-Specific Activated CD8 T Cells in E7 Transgenic Skin and Skin Grafts

**DOI:** 10.3389/fimmu.2017.00524

**Published:** 2017-05-04

**Authors:** Seyed Davoud Jazayeri, Paula T. Kuo, Graham Robert Leggatt, Ian H. Frazer

**Affiliations:** ^1^Diamantina Institute, University of Queensland, Brisbane, QLD, Australia

**Keywords:** human papillomavirus, skin grafting, E7-specific CD8+ T cells, T cell migration, immunotherapy

## Abstract

Human papillomavirus (HPV) 16 E7 (E7) protein expression in skin promotes epithelial hyperproliferation and transformation to malignancy. Grafts of murine skin expressing E7 protein as a transgene in keratinocytes are not rejected from immunocompetent recipients, whereas grafts expressing ovalbumin (OVA), with or without coexpression of E7 protein, are promptly rejected, demonstrating that E7-associated non-antigen-specific local immunosuppression is not a major determinant of lack of rejection of E7 transgenic skin. To determine whether failure of rejection of E7 skin grafts is due to failure to attract E7-specific effector T cells, E7- and OVA-specific effector CD8^+^ T cells, activated *in vitro*, were transferred to animals bearing E7 transgenic skin grafts. Three days after T cell transfer, E7-specific T cells were present in significantly greater numbers than OVA-specific T cells in the grafted skin on animals bearing recently placed or healed E7 grafts, without graft rejection, and also in the ear skin of E7 transgenic animals, without obvious pathology. E7 and OVA-specific T cells were present in lesser numbers in healed E7 grafts than in recently placed grafts and in lesser numbers in recently placed E7 transgenic epidermal grafts without E7-associated hyperproliferation, derived from E7 transgenic mice with a mutated *retinoblastoma* gene. These data demonstrate that effector T cells are to some extent attracted to E7 transgenic skin specifically by E7 expression, but in large measure non-specifically by the epithelial proliferation associated with E7 expression, and by the local inflammation produced by grafting. Failure of E7 graft rejection was observed despite trafficking of E7-specific effector T cells to E7-expressing epithelium, a finding of consequence for immunotherapy of HPV 16 E7-associated human cancers.

## Introduction

Infection with some human papillomavirus (HPV) genotypes promotes development of cervical intraepithelial neoplasia and cervical cancer. About 5% of all human cancers are caused by infection with high risk HPV genotypes, particularly HPV16, and HPV infection initiates more than 99% of cervical cancers, with over 260,000 related deaths (1). The annual global incidence of cervical cancer by the year 2050 is estimated to be more than 1 million (2). Although most high-risk HPV infections will be cleared within 5 years, infection is persistent in 1 to 2% of infected subjects and can progress to precancerous lesions and to cervical cancer if untreated (3, 4). Increased regulatory T cells in lesions correlate with virus persistence and cancer progression, while regressing lesions show a dominance of CD8+ T cell infiltrates (5–7).

A mouse model of epithelial pre-malignancy has been developed in which the HPV 16 E7 oncoprotein is expressed as a transgene within keratinocytes under the control of the keratin 14 transcriptional promoter (8). Expression of HPV type 16 E7 as a transgene in epidermis causes epidermal hyperplasia and induces a chronic inflammatory cell infiltrate, characteristics of pre-malignant lesions caused by HPV infection. The hyperplasia and inflammatory cell infiltrate are each dependent on interaction of E7 protein with retinoblastoma protein, as they are not present in E7 transgenic animals with a mutated *Rb* gene, giving rise to a functional Rb protein which is unable to bind E7 (K14E7.Rb^mut/mut^ mice) (9). E7 transgenic skin is not rejected when transplanted onto immune competent mice that can recognize the E7 as a non-self antigen (10, 11). Further, rejection of E7 transgenic skin grafts does not occur when E7-specific CD8 T cells are induced in graft recipients by immunization (12), by passive transfer of E7-specific T cells alone (11), or by placement of an E7 transgenic graft on an animal that has previously rejected an NKT cell-deficient E7 graft (13, 14). However, activation by immunization of large numbers of passively transferred E7-specific cytotoxic T cells will lead to rejection of recently placed but not well-healed E7 grafts, demonstrating that the local environment is a determinant of whether E7-specific cytotoxic T cells are attracted to E7-expressing skin and/or whether E7 is effectively presented by keratinocytes to those cells (11).

In the current study, we aimed to determine the efficacy of attraction of *ex vivo*-generated E7-specific CD8 T cells to E7 transgenic skin to test whether failure of attraction of antigen-specific T cells could explain the observed failure to reject E7-expressing grafts. We determined that *in vitro*-activated CD8 T cells, when transferred to animals bearing E7 grafts, are detected in the K14E7 graft and that E7-specific T cells are attracted in larger numbers than cells of an irrelevant specificity to the E7-expressing epidermis.

## Results

### Expression of E7 As a Transgene in Keratinocytes Does Not Prevent Rejection of Grafts Coexpressing Ovalbumin (OVA)

**Figure 1:**
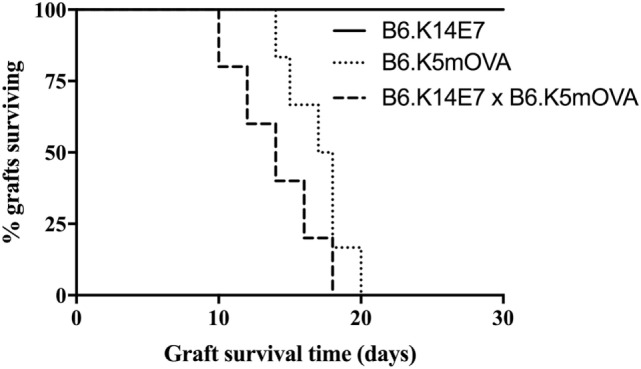
**Fate of skin grafts expressing ovalbumin (OVA) with or without E7 protein**. C57BL/6 (B6) mice received grafts of ear skin from B6.K14.E7, B6.K14.E7 × B6.K5mOVA, or B6.K5mOVA mice (*n* = 5 or 6 per group as shown). Curves show graft survival. The difference between B6.K14.E7 × B6.K5mOVA and B6.K5mOVA graft survival was non-significant by log-rank test.

Our previous studies have shown that grafted hyperproliferative E7 transgenic skin locally suppresses E7-specific CD8 T cell responses and graft rejection from an immunocompetent, non-transgenic graft recipient. Inhibition of rejection is mediated by IFN-γ and NKT cells in the hyperproliferative epithelium, as E7 transgenic grafts lacking IFNγR or NKT cells are rejected (13). The extent to which local immunoregulation of T effector functions is innate as opposed to antigen specific is currently unknown. To clarify this, we examined the fate of K14E7 skin grafts coexpressing OVA as a transgene from the K5 promoter, an antigen previously shown to induce skin graft rejection when expressed as a single transgene (15). K14 and K5 promoters each lead to transgene expression in basal keratinocytes. Consistent with our previously reported results, E7 skin grafts were not rejected from C57BL/6 mice (10, 11). In contrast, grafts from K5mOVA and K14E7 × K5mOVA animals were each rejected by day 18 post grafting (Figure 1), with no evidence of delayed rejection due to the coexpression of E7. This finding suggests that failure of rejection of an K14E7 graft by an immunocompetent recipient reflects antigen-specific failure of effector T cell response necessary for graft rejection. We also examined skin grafts from K14E7.Rb^mut/mut^ mice, where the Rb gene is mutated such that it cannot bind to the E7 protein and consequently the E7-expressing skin in these mice fails to induce epithelial hyperplasia. We observed that 5 of 5 K14E7.Rb^mut/mut^ freshly placed, skin grafts failed to reject, showing that the hyperplasia induced by E7–Rb binding is not the only reason for failure of E7 graft rejection. Therefore, we wished to determine whether failure of recruitment of antigen-specific effector T cells to the graft site might contribute to failure of graft rejection.

### Detection of Activated E7-Specific T Cells in the Epidermis of E7 Transgenic Skin Graft

**Figure 2:**
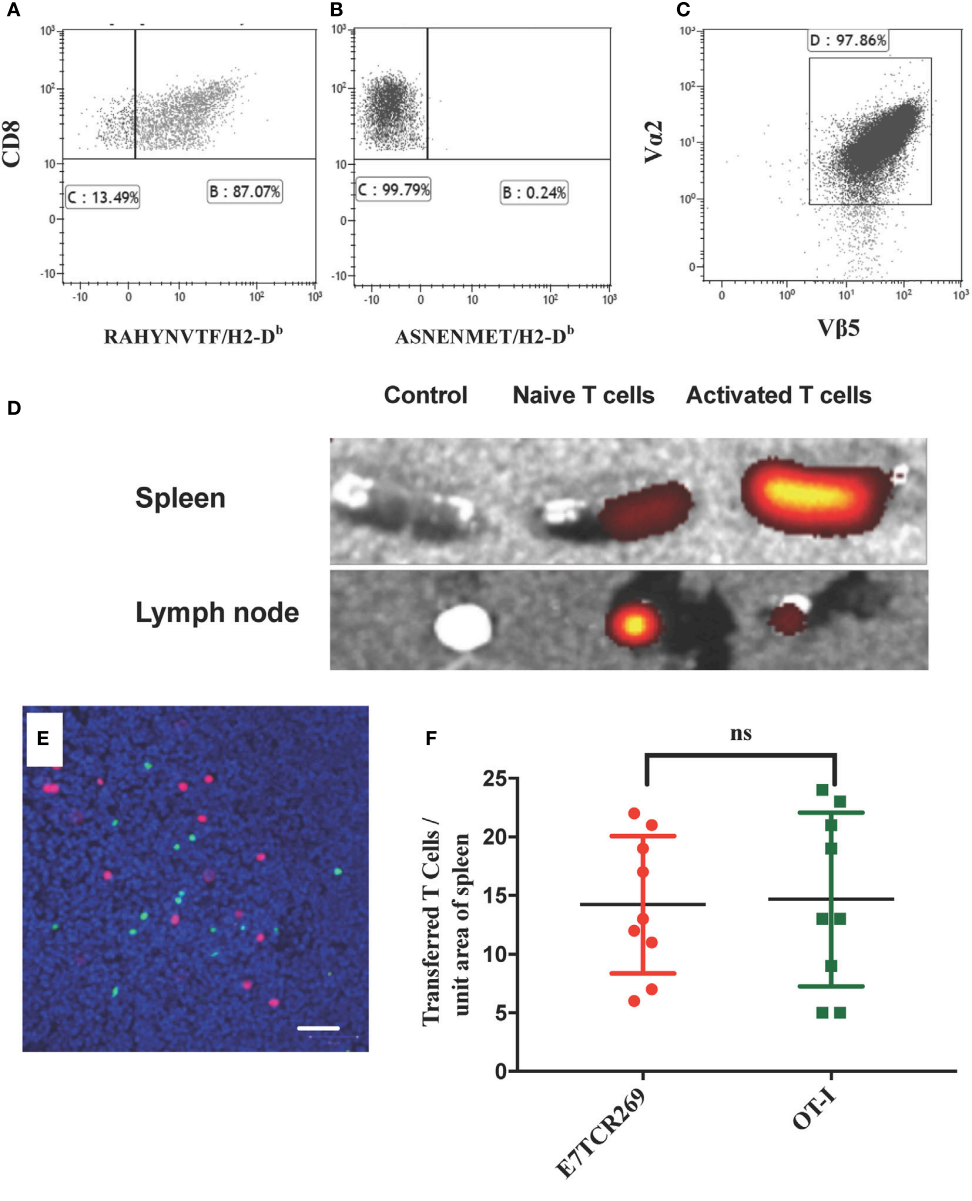
**Migration of naïve and activated antigen-specific T cells to lymphoid organs**. **(A,B)** Splenocytes from B6.E7TCR269 mice, transgenic for the β chain of an E7-specific T cell line, were stimulated *in vitro* for 2 weeks with an H2-D^b^-restricted peptide of E7 (RAHYNIVTF) and stained with a fluorescently labeled peptide/MHC dextramer consisting of **(A)** RAHYNIVTF/H2-D^b^ complexes or **(B)** ASNENMET/H2-D^b^ complexes. **(C)** Splenocytes from B6.OT-I mice, transgenic for a TCR specific for the ovalbumin peptide SIINFEKL, were stimulated *in vitro* for 2 weeks with SIINFEKL peptide. CD8 T cell expression of TCR Vα2 and Vβ5 is shown. Flow cytometry plots gated on live, singlet cells. **(D)** Spleen and inguinal lymph nodes were harvested from C57BL/6 mice 24 h after i.v. injection of 2.5 × 10^6^ fluorescently labeled (eFluor 670) CD8 T cells from B6.E7TCR269 mice, either naïve or exposed *in vitro* to E7 peptide as in **(C)**. Imaging of harvested organs by IVIS Spectrum Preclinical Imaging System. Control tissue was obtained from mice injected with PBS. **(E)** Equal numbers (2.5 × 10^6^ cells per mouse) of *in vitro*-activated E7TCR269 T cells labeled with eFluor 670 (green) and *in vitro*-activated OT-I CD8 T cells labeled with CytoPainter orange fluorescence (red) were injected intravenously into C57BL/6 mice. After 24 h, 10 µm frozen sections of spleen were prepared, counterstained with Hoechst 33342, and imaged by confocal Zeiss microscopy. Scale bar represents 50 µm. **(F)** Quantitation of cells from **(E)** over nine high power fields (ns = not significant).

K14E7 mice exhibit an extensive endogenous dermal lymphoid infiltrate of unknown specificity (9, 16). As failure of rejection of K14E7 grafts, and rejection of grafts expressing both E7 and OVA, was inconsistent with non-antigen-specific local immunosuppression of effector T cell function, we investigated whether activated E7-specific T cells could traffic to K14E7 skin and whether this trafficking was antigen specific. To examine antigen-specific T cells in the skin of K14E7 transgenic mice, we first established and validated lines of *ex vivo*-activated E7-specific and, as a control, OVA-specific T cells. Splenocytes from mice that are T-cell receptor transgenic for the β chain of an E7-specific T cell clone (E7TCR269 mice) (11) or splenocytes from OT-I mice with T cells specific for the SIINFEKL peptide of OVA (17) were stimulated *in vitro* with the H-2D^b^ restricted peptide derived from HPV16 E7 (RAHYNIVTF) or the H-2K^b^ peptide derived from OVA (SIINFEKL). After 2 weeks of *in vitro* restimulation with cognate peptide, 90% of E7TCR269 T cells specifically bound an H-2D^b^/RAHYNIVTF dextramer and not to an irrelevant dextramer (ASNENMET/H-2D^b^) (Figures 2A,B). After similar *in vitro* restimulation with SIINFEKL peptide, 97% of the OT-I CD8 T cells expressed the clonotypic Vα2/Vβ5 TCR (Figure 2C). Following injection of labeled activated T cells, these cells were present in the spleen of non-transgenic mice (Figure 2D) and when injected at a 1:1 ratio they were present in equal numbers (Figures 2E,F), validating the use of these lines to assess relative numbers of activated T cells in skin after transfer to E7 transgenic hosts or hosts bearing E7 transgenic skin grafts.

**Figure 3:**
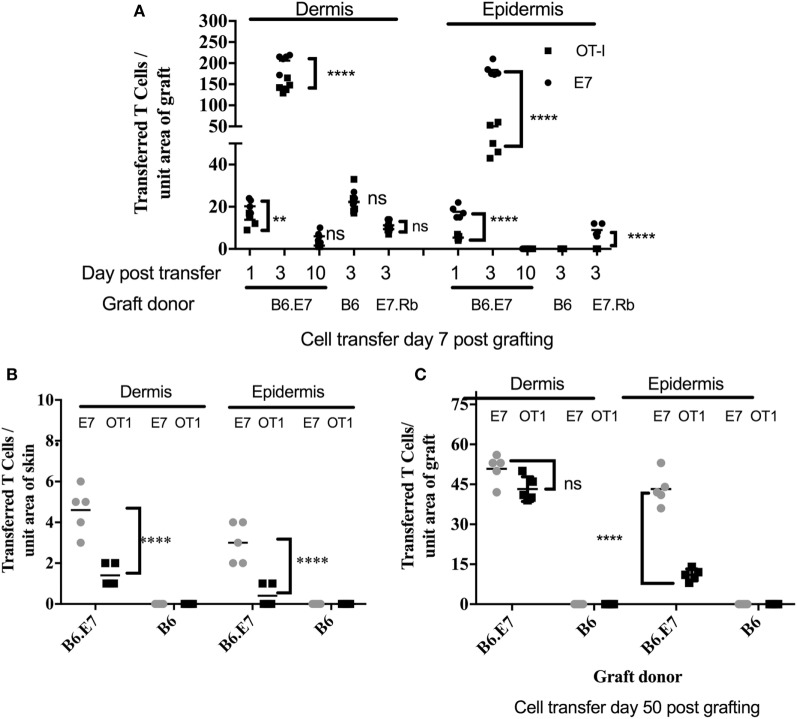
**Preferential migration of E7-specific CD8 T cells to the epidermis of hyperproliferative epithelial grafts and ear skin**. B6 mice received grafts of ear skin from B6, B6.K14.E7 (B6.E7), or B6.K14E7.Rb^mut/mut^ (E7.Rb) mice as shown. After 7 or 50 days, graft recipients received 2.5 × 10^6^ *in vitro* activated E7TCR269 (E7) and OT-1 CD8 T cells by i.v. injection, prepared and labeled as in Figure 2. At various time points after cell transfer, grafted **(A,C)** or donor ear **(B)** skin was harvested, frozen sections prepared, and the number of E7 and OT-1 cells enumerated in epidermis and dermis. **(A)** Transferred T cell numbers in B6, B6.E7, and E7.Rb grafts on B6 mice after cell transfer performed day 7 post grafting. **(B)** Transferred T cell numbers in ear skin of B6 and B6.E7 mice 3 days after cell transfer. **(C)** Transferred T cell numbers in B6 and B6.E7 grafts on B6 mice 3 days after cell transfer performed at day 50 post grafting. Data averaged over 35 areas of five independent grafting experiments are shown (****p < 0001, **p < 0.01; unpaired *t* test).

To examine T cell migration, E7-specific and OVA-specific activated T cell lines were first coadministered to mice recipient of E7 transgenic ear skin grafts (Figure 3A). Activated T cells were present in the dermis and the epidermis of E7 transgenic skin grafts placed 7 days previously, 1 day after T cell injection. T cells numbers were maximal at 3 days post injection and were almost undetectable by 10 days after injection (Figure 3A; Figure S1 in Supplementary Material). At day 3 post injection, transferred T cell numbers, regardless of antigen specificity, were significantly greater (*p* < 0.001, unpaired *t* test) in K14E7 grafts when compared with non-transgenic grafts (B6), in which there were equal numbers of E7-specific and OVA-specific activated T cells within the dermis (Figure 3A; Figures S1D,F in Supplementary Material) and none in the epidermis. At day 3, E7-specific T cell numbers were 3.6 times higher than OVA-specific T cells within the E7 transgenic epidermal grafts, whereas the ratio of E7 to OT-1 cells in the dermis of these grafts was 1.3 (Figure 3A). These data show that K14E7 skin grafts attract transferred, activated CD8 T cells of either specificity more effectively than non-transgenic skin grafts and that the epidermis of K14E7 skin preferentially attracts activated CD8 T cells with E7-specificity.

### E7-Associated Hyperplasia Results in Increased Numbers of Activated T Cells in the Skin

To determine whether E7-associated epithelial hyperproliferation contributed to accumulation of activated T cells in recently placed E7 transgenic grafts, mice expressing a mutated Rb gene (Rb^mut/mut^) (18) as well as the E7 transgene were used as donors of skin grafts expressing E7 protein without epithelial hyperplasia. Skin from mice doubly transgenic for K14E7 and mutant Rb (K14E7.Rb^mut/mut^) was grafted side by side with skin from K14E7 mice, and a mix of the labeled activated E7TCR269 and OT-I CD8 T cells at a 1:1 ratio was injected to graft recipients 7 days after grafting. While transferred CD8 T cells of both specificities were detected in K14E7 dermis and epidermis, the K14E7.Rb^mut/mut^ graft had significantly fewer transferred CD8 T cells than the K14E7 skin graft (Figure 3A), though still with greater numbers of E7-specific T cells as compared to OVA-specific T cells in the epidermis, where E7 but no OT-1 cells were seen, whereas in the dermis the numbers were approximately equal (ratio E7:OT-1 of 1.2) (Figure 3A; Figure S1E in Supplementary Material). These findings suggest that while E7 expression in skin attracts or retains E7-specific T cells preferentially to the epidermis, hyperproliferative epithelium also contributes to T cell attraction in a non-antigen-specific manner.

### Transferred, Activated CD8 T Cells Are Increased in K14E7 Ear Skin and in Healed K14E7 Grafts

Placement of skin grafts induces local inflammation which may contribute to the trafficking of antigen-specific CD8 T cells to the skin. To determine accumulation of transferred CD8 T cells in the skin in the absence of graft-induced inflammation, we quantitated accumulation of transferred cells to ear skin of K14E7 mice and also to well-healed K14E7 grafts at day 50 when markers of inflammation had resolved. In both K14E7 skin and well-healed K14E7 skin grafts, the numbers of transferred cells in skin were reduced when compared with numbers of transferred T cells within grafts placed 7 days prior to cell transfer (Figures 3B,C). Transfer of CD8 T cells to a K14E7 mouse was expected to result in dilution of cells throughout the entire mouse skin and thus cell accumulation per area of skin could not be directly compared with cells numbers attracted to a graft. However, the total absence of transferred, activated cells in non-transgenic animal skin and the larger numbers of E7-specific cells compared with OT-1 cells in the skin of E7 transgenic animals 3 days after T cell transfer confirms that local antigen encourages accumulation of antigen-specific effector T cells. Similarly, the restriction of E7-specific T cells to well-healed E7 transgenic and not to non-transgenic grafts (Figure 3C) and the greater numbers of E7 specific T cells as opposed to OT-1 cells in the epidermis (ratio E7:OT-1; 78.5) as opposed to the dermis (ratio E7:OT-1; 3.1) in the E7 transgenic grafts, confirms the findings with recently placed grafts, while confirming that inflammation also contributes to non-antigen-specific promotion of T cell trafficking.

### Activated CD8 T Cells Migrate to K14E7 Skin Grafts More Effectively than Naïve T Cells

**Figure 4:**
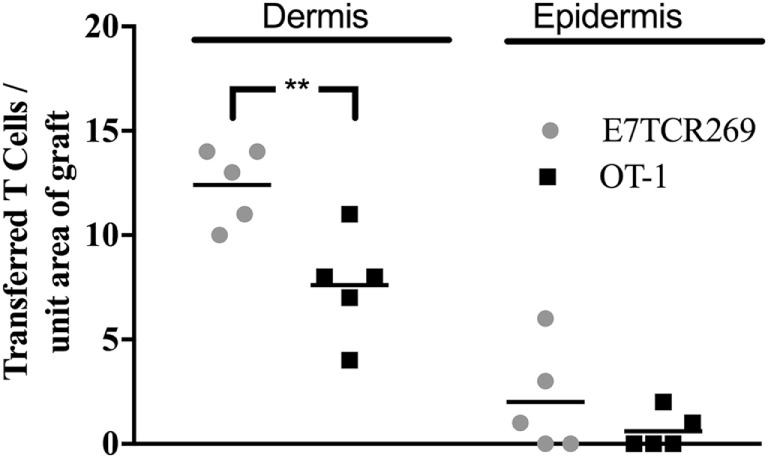
**Migration of naïve TCR transgenic CD8 T cells to grafted K14E7 ear skin**. C57BL/6 mice received ear skin grafts from B6.K14.E7 mice. After 7 days, graft recipients received 2.5 × 10^6^ naïve E7TCR269 and OT-1 CD8 T cells by i.v. injection, labeled as in Figure 2. At 3 days after transfer, graft skin was harvested, frozen sections prepared, and the number of E7TCR269 and OT-1 CD8 T cells enumerated in epidermis and dermis. Data averaged over 35 areas of five independent grafting experiments are shown (**p < 0.05, ns = not significant; unpaired *t* test).

To determine whether E7-specific CD8 T cells need to be activated *ex vivo* to migrate to hyperplastic E7 transgenic grafted skin, E7TCR269 CD8 T cells and OT-I CD8 T cells were isolated from spleen, fluorescently labeled, and transferred without *in vitro* activation to mice recipient 7 days previously of K14E7 and non-transgenic skin grafts. At day 3 post injection, labeled T cells were enumerated in K14E7 and non-transgenic grafts. Only a few naïve E7TCR269 and OT-I CD8 T cells were observed in the epidermis and dermis of the K14E7 grafts (Figure 4), when contrasted with the findings with activated T cells, confirming that CD8 T cells, whether antigen specific or otherwise, need to be activated to appear in the grafts within 3 days. The results from this study, when taken together, show that hyperplastic skin, and recently grafted skin, attract transferred activated T cells more effectively than naïve T cells and that E7-specific activated T cells are detected in larger numbers in E7 transgenic epidermis than activated T cells of other specificity. Activated E7-specific T cells can thus localize to K14E7 skin (or skin grafts), and failure of K14E7 grafts to reject is not likely to reflect failure of trafficking of antigen-specific cells to the E7 transgenic skin, implying that the mechanism of antigen-specific suppression of immune effector function observed with E7 skin is more likely to be local regulation of effector T cell function than a hindrance to migration of E7-specific effector cells.

## Discussion

In this paper, we show that activated T cells specific for the HPV16 E7 protein traffic effectively to epidermis expressing the HPV16 E7 protein as a transgene in keratinocytes. We show further that while activated T cells migrate non-specifically to hyperproliferative epithelium as previously shown (9) there are more E7-specific T cells than irrelevant specificity T cells within the epidermis, both in non-injured E7 transgenic skin and in grafted E7 transgenic skin. However, transfer of E7 antigen-specific T cells to animals bearing E7 transgenic skin grafts is nevertheless unable to induce rejection of those grafts. This finding is consistent with the inability of E7 immunization to effect rejection of E7-expressing skin in mice (12) or in humans (4), despite the ability of such immunization to enable antigen-specific rejection of established E7-expressing transplantable tumors in mice in many studies (3). Thus, failure of E7-expressing keratinocytes to encounter E7 antigen-specific effector T cells at the site of antigen expression is not the sole explanation for the observed lack of rejection of E7-expressing skin grafts, a finding that supports the hypothesis that there is local antigen-specific immunoregulation. An alternate explanation to local immunoregulation as an explanation for the observed findings would be that presentation of E7 protein derived peptides by KC to effector T cells is insufficient to trigger T cell effector functions. This explanation is considered unlikely as K14 E7 transgenic skin grafts are rejected when substantial numbers of T cells TCR transgenic for the beta chain of an E7 peptide specific TCR are transferred to a graft recipient, and the graft recipient is subsequently immunized with E7 protein (11), and also as K14E7 grafts from a wide range of donors and/or recipients genetically manipulated to remove immunoregulatory components of the immune system (NKT cells, IFNγ, Eosinophils, Mast cells, IDO, IL-1RA) are rejected even without E7-specific immunization of the recipient animal (4, 14, 16, 19).

Initiation of tumors not only requires genetic and epigenetic alterations to cell cycle control but also the generation of a supportive microenvironment (20). One important component of the tumor microenvironment is recruited components of the innate immune system, which can either promote or detract from tumor growth, and can also regulate the function of local antigen-specific immune T cells. Although inflammation in cancer has generally focused on the activity of innate immune cell responses, the presence of infiltrating lymphocytes has also been recognized in solid tumors (21). Accumulation of a T cell-enriched inflammatory infiltrate in E7-expressing skin is characteristic of pre-malignant lesions associated with the HPVE7 transgene (9). Hyperplasia associated with E7 expression is likely partly responsible for the accumulation of T cells in human HPV-associated cancers, as the hyperplastic malignant epithelium expresses increased levels of chemokines (22) as is also seen in the mouse (16). Further support for this hypothesis is provided by the observation that non-hyperplastic E7 transgenic skin of the K14E7.Rb^mut^ mouse line does not attract a significant inflammatory infiltrate or express elevated chemokine mRNAs (23).

Placement of E7-expressing grafts lacking lymphocytes (9), NKT cells (14), or IFN-γ (19) results in CD8 T cell-dependent (11) rejection of the genetically manipulated E7 transgenic graft, but not of a neighboring E7 transgenic but otherwise unmanipulated graft. Thus, tolerance of grafted E7 skin cannot be due to failure of keratinocytes to present E7 to the immune system locally in the graft. Rejection of the variously genetically manipulated E7 transgenic skin grafts shows that E7 is presented effectively from E7 skin both for induction of E7-specific T cell responses and also to allow recognition of the graft by effector T cells, and thus local effector T cell function must be regulated locally by a cell or cells of the adaptive immune system, likely NKT cells (14), or by the cytokines they secrete including IFNγ (13). These local regulatory processes, induced by E7, do not suppress rejection by OVA-specific CD8 T cells of a graft expressing both E7 and OVA. Paradoxically, however, grafts from K14E7.Rb^mut^ mice expressing E7 without hyperplasia or the hyperplasia-associated local inflammatory infiltrate are not rejected following immunization with E7, or by passive transfer of T cells, and fail to attract large numbers of E7-specific effectors (24), suggesting that local inflammation induced by E7 is necessary for immune effector function, and that components of the inflammatory infiltrate must both assist and hinder effector T cell-mediated rejection of E7 transgenic skin. E7 transgenic keratinocytes express comparable endogenous levels of E7 protein to a range of CTL-sensitive E7-expressing cell lines (25) but are not susceptible to CTL-mediated lysis *in vitro*, though E7 transgenic and non-transgenic keratinocytes are susceptible to conventional mechanisms of CTL-mediated lysis, including perforin and Fas/FasL interaction, when an excess of exogenous peptide is provided. Thus, a component of the inflammatory environment, possibly IFNγ (26) likely upregulates the presentation of E7 by keratinocytes to effector T cells, while another component, perhaps also IFNγ, inhibits effector T cell function, possibly by inducing PD-L1 expression locally, or by directly or indirectly interfering with effector T cell survival or maturation in an antigen-specific manner.

The chemokines controlling lymphocyte trafficking to HPV skin are under investigation with one report suggesting that Th17 cells infiltrate human cervical cancer *via* CCR6/CCL20 interactions (27). In several skin conditions, CCR4, CCR6, CCR8, and CCR10 have been implicated in the trafficking of T cells to skin tissue (28). Moreover, Tan et al. (28, 29) showed, Temozolomide (TMZ), a chemotherapeutic drug used to treat metastatic melanoma, induced T cell infiltration into transplanted melanoma and into genitourinary tumors in mice developing spontaneous melanoma. In contrast, TMZ treatment did not increase T-cell infiltration into cutaneous tumors, despite similar increases in the expression of the chemokines CXCL9 and CXCL10 in all sites after TMZ exposure.

Our study indicates that activated CD8 T cells are generally attracted to K14E7 transgenic skin, regardless of antigen specificity. However, once in the skin, E7-specific T cells were seen to show greater localization in the epidermis relative to OT-1 cells, suggesting that antigen-specific signals within the epidermis might favor the recruitment or retention of E7-specific cells. Studies using Herpes Simplex Virus infection of skin suggest that a population of CD8 T cells can become resident within the epidermis (tissue-resident memory T cells or Trm) and that cells are retained or maintained there through expression of CD69, CD103 and responsiveness to IL-15 (30, 31). Interestingly, the development of Trm can also occur in the absence of an antigen-specific interaction, as inflammation was able to direct OVA-specific and HSV-specific CD8 T cell development into Trm within the epidermis (32). Recently, Khan et al. (33) infected the skin with vaccinia virus (VacV) and found activated CD8+ T cells trafficked to VacV-infected skin in an inflammation-dependent, but antigen-independent manner. However, after viral clearance, when antigen still present in the tissue microenvironment, there was a subsequent ~50-fold increase in Trm formation. Moreover, secondary antigen stimulation caused CD8+ T cells to rapidly express CD69 and be retained at the site of infection (33). Expression of the chemokine receptor, CCR6, was found to be enriched among CD4 T cells that infiltrated K14E7 skin although the dermal or epidermal location of these cells was not established (9). The transferred CD8 T cells in our study were not retained long term, suggesting that conventional Trm did not develop. However, the failure to develop Trm may reflect the *in vitro* culture system used in the current study where the development of short-lived, IL-2-dependent effector T cells may have been favored over memory T cell precursors. Both the development of resident memory T cells and the chemokines which attract E7-specific CD8 T cells to the K14E7 transgenic epidermis are currently under investigation within our group.

## Materials and Methods

### Mice

C57BL/6, HPV16-E7-transgenic C57BL/6 mice (K14.E7) (8), and K5mOVA transgenic mice were obtained from the Animal Resources Centre (ARC, Perth, WA, Australia). K14.E7^+/−^ × Rb9^−/−^ (34), mice (K14E7.Rbmut) were provided by Paul Lambert lab, Madison, Wisconsin, USA. E7TCR269 were generated in our laboratory by G. Leggatt (35) and OT-I mice were purchased from The Jackson Laboratories (Bar Harbor, ME, USA). K5mOVA mice were bred with K14.E7 mice to produce mice heterozygous for both transgenes.

All mice were kept under specific pathogen-free conditions at the Biological Research Facilities of Translational Research Institute and were sex matched and used at 6–12 weeks of age. Ear to flank skin grafts (groups of five mice) and assessment of graft outcomes to 50 days post grafting were performed as previously described (16).

### Ear Skin Grafting

Donor ear skin was grafted onto recipient flanks as previously described (16). Briefly, dorsal and ventral surfaces of ear skin from donor mice were placed onto the left thoracic flank region of an anesthetized recipient. Grafts were held in place with antibiotic-permeated gauze (Bactigras; Smith and Nephew, London, UK) and bandaged with micropore tape and Flex-wrap (Lyppard, QLD, Australia) for 7 days, and assessed as technically successful if they were adherent and vascularized on day 7. Graft rejection was assessed by a loss of distinct border and signs of ulceration and/or necrosis to >80% of the graft area (16).

### Reagents and Flow Cytometry

Anti-mouse monoclonal antibodies were obtained to CD45 (104), CD8 (SK1), Vβ5 (MR9-4) BioLegend (San Diego, CA, USA), CD16/32 (2.4G2) BD Pharmingen (SanDiego, CA, USA), Vα2 (B20.1) BD Pharmingen (San Diego, CA, USA), and Dextramers specific for RAHYNVTF/H2-D^b^ or for an irrelevant TCR ASNENMET/H2-D^b^ (Immudex). Cell suspensions were incubated with mAbs to CD16/32 for 30 min, followed by Live/Dead Fixable Aqua Dead Cell Stain (Invitrogen) at 4°C to block unspecific staining and exclude dead cells. All mAbs were incubated for 30 min at 4°C at predetermined optimal concentrations. Flow cytometric analysis was performed using the LSRII (BD Biosciences). Data were analyzed using the Kaluza software 1.2 (Beckman Coulter). Doublets and dead cells were excluded from analysis.

### *In Vitro* Activation of E7TCR269 and OT-I CD8 T Cells

Splenocyte suspensions from E7TCR269 and OT-I mice were depleted of red blood cells with H-2 ACK lysis buffer (0.15 MNH_4_Cl, 1 mM KHCO_3_, and 0.1 mM EDTA) and plated at 7.5 × 10^6^ cells per well in a 24-well plate. Syngeneic splenocytes were pulsed for 2 h with 0.01 µM of H-2^b^-restricted HPV16-E7 peptide (RAHYNIVTF) or OVA peptide (SIINFEKL) and gamma irradiated (3,000 rad) and were added at 3.5 × 10^6^ cells per well with 1 ng/mL (approximately 10 U/mL) recombinant mouse interleukin 2 (rIL-2) (BD Pharmingen, San Diego, CA, USA) in complete RPMI 1640 medium [RPMI supplemented with 0.216 g/L l-glutamine, 60 mg/L benzyl penicillin (CSL, Melbourne, VIC, Australia), 100 mg/L streptomycin (CSL), 50 µmol/L 2-mercaptoethanol, and 10% fetal bovine serum (FBS; Gibco, Gaithersburg, MD, USA)]. Splenocytes were then cultured at 37°C in a 5% CO_2_ incubator. After 1 week, 0.5 × 10^6^ of the resultant T cell lines were further cultured with 5 × 10^6^ irradiated, syngeneic splenocytes pulsed with RAHYNIVTF or SIINFEKL. T cell lines were harvested at day 14 and analyzed by flow cytometry using anti-CD8 antibody and Dextramers of RAHYNIVTF/H2-D^b^ complexes or for ASNENMET/H2-D^b^ (E7 lines) or for expression of CD8, Vα2, and Vβ5 (OVA lines). Cells viability was determined by Bio-Rad TC20 Cell Counter.

### Isolation of Naïve E7-TCR and OT-I Transgenic CD8 T Cells

CD8 T cells were isolated from pooled splenocytes of E7TCR269 or OT-1 mice using an EasySep Mouse CD8-T Cell Enrichment Kit (StemCell Technologies) according to manufacturer’s instructions. Cell viability was determined by Bio-Rad TC20 Cell Counter and purity of isolated cells were determined by FACS.

### CD8 T Cells Labeling and Tail Vein Injection (i.v.)

*In vitro* activated and naïve E7TCR269 and OT-I CD8 T cells were harvested, washed, filtered, and labeled with eFluor 670 (eBioscience) or CytoPainter orange fluorescence (ab176737) according to manufacturer’s instructions. Labeled E7TCR269 and OT-I CD8 T cells were resuspended at a 1:1 ratio in sterile PBS and injected intravenously.

### Fluorescence Imaging Studies

#### Real-time Fluorescence Imaging

Spleen, inguinal, and axillary lymph nodes and skin was harvested at various times after injection of labeled cells and imaging was carried out using the IVIS Spectrum Preclinical Imaging System. Alternatively, unfixed 35-µm frozen tissue sections were counterstained with Hoechst 33342 and imaged by confocal Zeiss microscopy, and the absolute number of labeled E7TCR269 and OT-I CD8 T cells per unit area were counted in five random selected fields (350 μm × 350 μm) of seven different sections.

### Statistical Analysis

Prism (GraphPad Software, La Jolla, CA, USA) software was used to prepare graphs and for statistical analysis. Statistical analysis was carried out using 2-way analysis of variance or an unpaired *t* test as shown. Error bars on graphs represent SEM.

## Ethics Statement

All animal procedures and experiments were performed in compliance with the ethical guidelines of the National Health and Medical Research Council of Australia, with approval from the University of Queensland Animal Ethics Committee (#367-13).

## Author Contributions

SJ and IF conceived the study. SJ conducted the experiments and wrote the manuscript. All authors analyzed the results and reviewed the manuscript.

## Conflict of Interest Statement

The authors declare that the research was conducted in the absence of any commercial or financial relationships that could be construed as a potential conflict of interest.
