# Supplementary material for: HPV16-E7-Specific Activated CD8 T Cells in E7 Transgenic Skin and Skin Grafts
Source: Front Immunol. 2017 May 4;8:524. doi: 10.3389/fimmu.2017.00524 (PMC5415560; doi:10.3389/fimmu.2017.00524)
Supplement: Supplementary file 1 [file Image_1.PDF]

Supplementary Figure 1

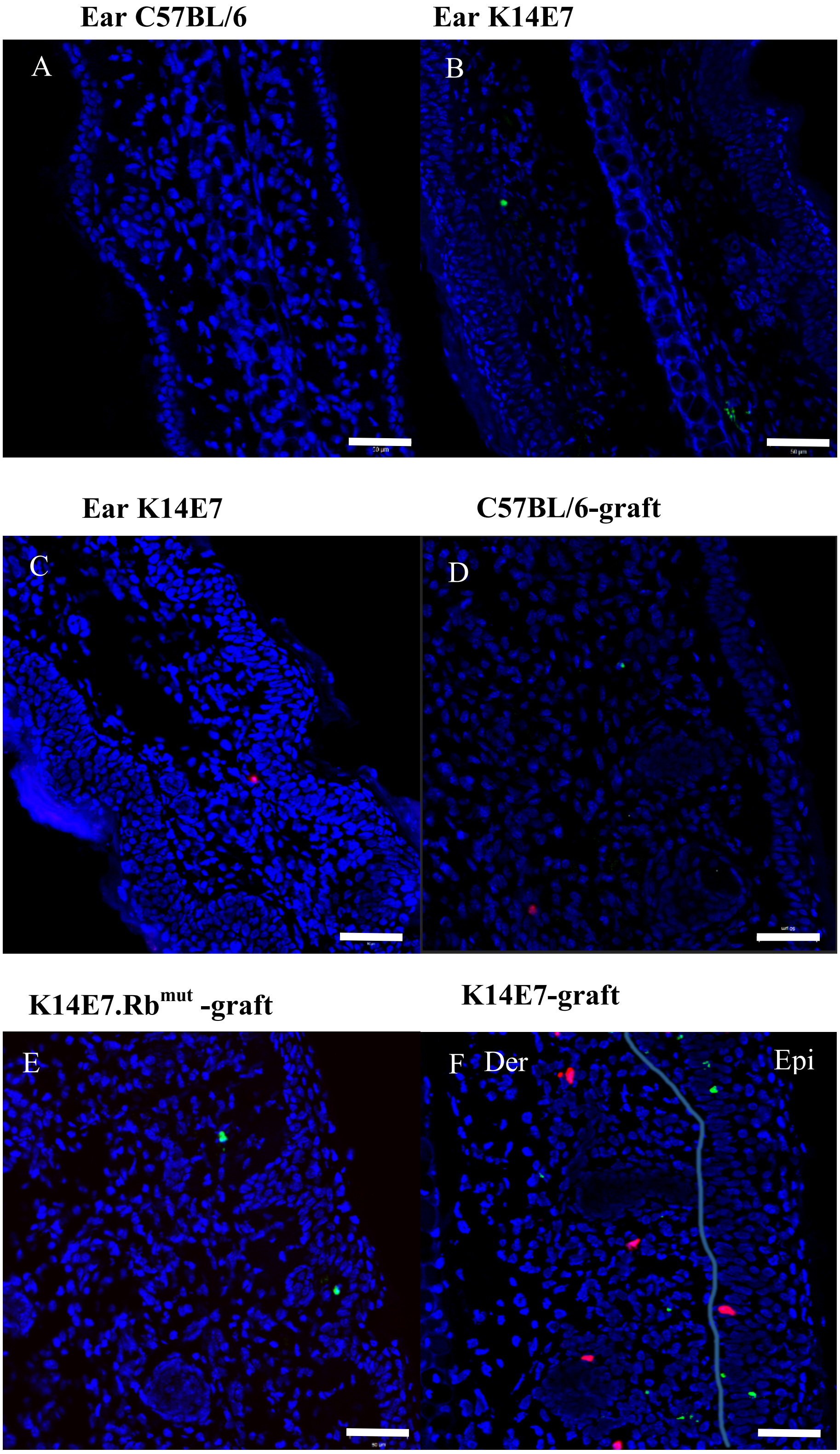

Supplementary Figure 1 Preferential migration of E7 specific CD8 T cells to epidermis

(**A**) C57BL/6 mice and (**B, C**) K14E7 mice received 6×10^6 *in vitro* activated E7TCR269 (Green) and OT-I (Red) CD8 T cells by iv injection. C57BL/6 mice were grafted with ear skin from (**D**) C57BL/6, (**E**) K14E7.$Rb^{mut/mut}$ or (f) K14E7 mice, and received 2.5×10^6 of *in vitro* activated E7TCR269 (Green) and OT-I (Red) CD8 T cells by iv injection. Unfixed sections (35 µm) of (**A, B, C**) ear or (**D, E, F**) ear graft skin was counterstained with Hoechst dye and imaged by confocal UV microscopy. Scale bar represents 50µm.
